# Transplate Cardíaco e a “Câmara Secreta”: Como a Avaliação Ecocardiográfica do Ventrículo Direito pode Revelar a Rejeição Celular Aguda

**DOI:** 10.36660/abc.20200177

**Published:** 2020-05-12

**Authors:** Henrique Turin Moreira, Minna Moreira Dias Romano

**Affiliations:** 1 Faculdade de Medicina de Ribeirão Preto USP Ribeirão Preto SP Brasil Faculdade de Medicina de Ribeirão Preto USP, Ribeirão Preto, SP – Brasil

**Keywords:** Rejeição Celular Aguda, Transplante do Coração, Biópsia Endomiocárdica, Rejeição do Enxerto, Ecocardiografia/métodos

Embora o transplante (Tx) cardíaco tenha alcançado grande evolução técnica e científica nas últimas décadas, a rejeição celular aguda (RCA) ainda representa uma importante ameaça aos pacientes submetidos a esse procedimento. Protocolos de rastreio de rejeição aguda associados à terapêutica imunossupressora precoce são essenciais para o sucesso do mesmo. No entanto, a biópsia endomiocárdica, embora dispendiosa e invasiva, ainda é método de referência para o rastreio da RCA.

A RCA relaciona-se a danos incipientes na função miocárdica,^1^ que podem não ser detectados pelas técnicas ecocardiográficas convencionais de análise da função miocárdica. A deformação miocárdica, analisada pela técnica de rastreamento de pontos, é capaz de detectar disfunção miocárdica incipiente em diversas patologias, dentre elas, a RCA pós Tx cardíaco.^2^

Em recente metanálise envolvendo 10 estudos com semelhanças metodológicas, Elkaryone et al.,^3^ analisaram 511 pacientes e 1.267 biópsias endomiocárdicas. A sensibilidade da deformação longitudinal global do ventrículo esquerdo (VE), expressa pelo GLS, para detectar RCA diagnosticada por biópsia endomiocárdica foi de 78%, com especificidade de 68%.^3^ Ademais, as alterações de deformação miocárdica do VE já foram demonstradas como preditores independentes de desfechos clínicos após o Tx cardíaco.^4^ Importante ressaltar que nem sempre as imagens ecocardiográficas permitem a análise de deformação miocárdica nesta população, uma vez que o coração transplantado pode estar em posição mais medial na cavidade torácica, dificultando a obtenção de imagens de boa qualidade, como previamente demonstrado.^2^ No entanto, a despeito de estudos das alterações de VE neste cenário, menos conhecimento foi acumulado até o momento atual acerca das correlações entre alterações do ventrículo direito (VD) e RCA após Tx cardíaco. É reconhecido o aumento das dimensões desta câmara associado à leve redução de sua função sistólica na evolução natural de pacientes após o Tx cardíaco.^5 - 7^

O trabalho de Carrion et al.,^8^ demonstram que pacientes com sinais de RCA significativa pela biópsia endomiocárdica apresentam redução de parâmetros de função diastólica ventricular esquerda, além de sinais de aumento da espessura da parede posterior do VE, comparados aos sem rejeição significativa. Divergente de outros estudos, não houve diferença significativa da deformação longitudinal do VE entre este grupo e aquele que não apresentou sinais de rejeição significativa. Vale ressaltar que a maioria dos estudos prévios usou, para análise da deformação miocárdica, *softwares* vendedores-dedicados, enquanto Carrion et al.,^8^ usaram *software* independente de vendedor na análise das imagens,^8^ o que poderia explicar, pelo menos em parte, as divergências citadas, uma vez que ainda não há padronização entre *softwares* de diferentes vendedores.^8^

Outra possível fonte de divergência entre o presente estudo e investigações prévias é a metodologia empregada para a medida da deformação miocárdica. Carrion et al.,^8^ realizaram a medida do pico sistólico da deformação miocárdica, assim como recomendado pelas mais recentes diretrizes internacionais,^9^ enquanto outros trabalhos utilizaram o pico de deformação miocárdica de todo o ciclo cardíaco.^9^ Ainda, o *software* utilizado para análises por Carrion et al.^8^ baseia-se na análise da deformação endocárdica, enquanto outros *softwares* disponíveis, e historicamente mais utilizados, analisaram a deformação miocárdica de toda a espessura da parede, incluindo todas as suas camadas, também chamada deformação transmural.^10^ Neste estudo os parâmetros ecocardiográficos convencionais de análise da função do VD, assim como a deformação miocárdica do VD, apresentaram-se significativamente reduzidos no grupo de pacientes com sinais de RCA em comparação com aqueles sem rejeição significativa.11 Além disso, o grupo com RCA moderada também mostrou alterações sugestivas de pior função diastólica do VE em comparação com o grupo sem rejeição significativa.

Dessa forma, permanece a dúvida se o envolvimento da função sistólica do VD é primário, devido à RCA, ou secundário ao aumento retrógrado das pressões de enchimento do VE. Além disso, não apenas a deformação miocárdica do VD, mas também os parâmetros convencionais de avaliação funcional dessa câmara cardíaca mostraram-se significativamente diferente entre os dois grupos estudados por Carrion et al.,^8^ Estudos prévios demonstram que a deformação sistólica do VD, especialmente de sua parede livre, apresenta maior correlação com a função sistólica do VD, avaliada por métodos de referência, em comparação com parâmetros ecocardiográficos convencionais, tanto em cardiopatias isquêmicas, quanto em não-isquêmicas.^12 - 14^ Embora a comparação da acurácia diagnóstica dos parâmetros funcionais do VD para diagnóstico da RCA não tenha sido abordada por Carrion et al.,^8^ essa questão é ponto relevante a ser esclarecido em estudos futuros, para desse modo nortear o emprego dessas técnicas na rotina clínica pós Tx cardíaco.

Assim, para uma ciência que há pouco tempo conseguiu reconhecer e “apontar” para a “câmara secreta”, o VD, o trabalho de Carrion et al.,^8^ reforça a importância da avaliação ecocardiográfica da mesma para a detecção não invasiva da disfunção miocárdica incipiente relacionada à RCA em pacientes em seguimento após Tx cardíaco.
