# Supplementary material for: Transplate Cardíaco e a “Câmara Secreta”: Como a Avaliação Ecocardiográfica do Ventrículo Direito pode Revelar a Rejeição Celular Aguda
Source: Arq Bras Cardiol. 2020 May 12;114(4):645–6. [Article in Portuguese] doi: 10.36660/abc.20200177 (PMC9744335; doi:10.36660/abc.20200177)
Supplement: Translation

# Translation: Heart Transplantation and the “The Secret Chamber”: How Echocardiographic Assessment of the Right Ventricle Can Reveal Acute Cell Rejection

**Authors:** Henrique Turin Moreira, Minna Moreira Dias Romano

**Affiliations:** 1 Brazil Faculdade de Medicina de Ribeirão Preto USP, Ribeirão Preto, SP – Brazil

**Keywords:** Cellular Acute Rejection, Heart Transplantation, Endomyocardial Biopsy, Graft Rejection, Echocardiography/methods

Although heart transplantation (Tx) has achieved great technical and scientific evolution in recent decades, acute cell rejection (ACR) still represents an important threat to patients submitted to this procedure. Acute rejection screening protocols associated with early immunosuppressive therapy are essential for Tx success. However, endomyocardial biopsy, although expensive and invasive, is still a reference method for the screening of ACR.

ACR is related to incipient damage in myocardial function,^1^ which may not be detected by conventional echocardiographic techniques for myocardial function analysis. Myocardial deformation, analyzed by the speckle tracking technique, is able to detect incipient myocardial dysfunction in several pathologies, among them, ACR after cardiac Tx.^2^

In a recent meta-analysis of 10 studies with methodological similarities, Elkaryone et al.^3^ analyzed 511 patients and 1,267 endomyocardial biopsies. The sensitivity of the global longitudinal deformation of the left ventricle (LV), expressed by the GLS, to detect ACR diagnosed by endomyocardial biopsy was 78%, with a specificity of 68%.^3^ Moreover, changes in LV myocardial deformation have already been demonstrated as independent predictors of clinical outcomes after heart Tx.^4^ It is important to note that echocardiographic images do not always allow the analysis of myocardial deformation in this population, since the transplanted heart may be in more medial position in the thoracic cavity, making it difficult to obtain good quality images, as previously demonstrated.^2^ However, despite studies of LV changes in this scenario, less knowledge has been accumulated to date about the correlations between changes in the right ventricle (RV) and ACR after heart Tx. The increase in the dimensions of this chamber associated with the slight reduction of its systolic function in the natural evolution of patients after cardiac Tx has been established.^5 - 7^

The work by Carrion et al.^8^ demonstrate that patients with signs of significant ACR by endomyocardial biopsy have reduced left ventricular diastolic function parameters, in addition to signs of increased LV posterior wall thickness, when compared to those without significant rejection. Unlike other studies, there was no significant difference in the LV longitudinal deformation between this group and the one that did not show signs of significant rejection. It is worth mentioning that most of the previous studies used, for the analysis of myocardial deformation, vendor-dedicated software, while Carrion et al.^8^ used vendor-independent software to analyze the images,^8^ which could explain the aforementioned differences, at least in part, since there is still no standardization between software from different vendors.^8^

Another possible source of divergence between the present study and previous investigations is the methodology used to measure myocardial deformation. Carrion et al.^8^ performed the measurement at the systolic peak of myocardial deformation, as recommended by the most recent international guidelines,^9^ while other studies used the peak myocardial deformation of the entire cardiac cycle.^9^ Also, the software used for the analysis by Carrion et al. is based on the analysis of endocardial deformation, while other software available, and historically more often used, analyzed the myocardial deformation of the entire wall thickness, including all its layers, also called transmural deformation.^10^ In this study, the conventional echocardiographic parameters for the analysis of the RV function, as well as the RV myocardial deformation, were significantly reduced in the group of patients with signs of ACR, when compared to those without significant rejection.^11^ Moreover, the group with moderate ACR also showed changes suggestive of worse LV diastolic function, when compared to the group without significant rejection.

Thus, the question remains whether the involvement of RV systolic function is a primary one, due to ACR, or secondary to the retrograde increase in LV filling pressures. Additionally, not only the myocardial deformation of the RV, but also the conventional parameters for the functional evaluation of this cardiac chamber have shown be significantly different between the two groups studied by Carrion et al.^8^ Previous studies have shown that RV systolic deformation, especially of its free wall, has a greater correlation with RV systolic function, assessed by reference methods, in comparison with conventional echocardiographic parameters, both in ischemic and non-ischemic heart diseases.^12 - 14^ Although the comparison of the diagnostic accuracy of the RV functional parameters for ACR diagnosis has not been addressed by Carrion et al.,^8^ this issue is a relevant point to be clarified in future studies, aiming to guide the use of these techniques in the post-heart Tx clinical routine.

Thus, for a science that recently managed to recognize and “point” to the “chamber of secrets”, i.e., the RV, the work of Carrion et al.^8^ reinforces the importance of its echocardiographic evaluation for the non-invasive detection of incipient myocardial dysfunction related to ACR in patients being followed after heart Tx.
